# Generative AI as a bounded elicitation and evidence-synthesis layer for pain assessment in older adults with cognitive impairment

**DOI:** 10.3389/fpain.2026.1922289

**Published:** 2026-10-28

**Authors:** Anitha Saravanan, Will Lewis, Jinbing Bai, Staja Booker

**Affiliations:** 1 Northern Illinois University, DeKalb, IL, United States; 2 Illinois State University, Normal, IL, United States; 3 Emory University, Atlanta, AT, United States; 4 University of Florida, Gainesville, FL, United States

**Keywords:** cognitive impairment, dementia, generative artificial intelligence, older adults, pain assessment

## Abstract

Pain assessment in older adults with cognitive impairment remains challenging, particularly when changes in communication make self-report difficult. Validated observational tools, caregiver and staff knowledge, clinical examination, and repeated within-person assessment remain the foundation of care. However, information from these sources may be incomplete, conflicting, or documented across different settings. Generative artificial intelligence (AI), including large language models, may help clinicians organize and review this information, but it should not independently detect, diagnose, or determine treatment for pain. In this Perspective article, we propose a person-centered, clinician-governed framework for the bounded use of generative AI in pain assessment. The framework organizes seven testable use cases under three core functions: capacity-sensitive elicitation, source-attributed synthesis, and longitudinal support and communication. Potential applications include adaptive pain interviews, identification of possible pain-related information in everyday language, structuring of behavioral observations, multi-informant synthesis, synthesis of validated nonverbal indicators, longitudinal pain-related diaries, and caregiver communication support. Each application requires clear limits, source identification, communication of uncertainty, consideration of alternative causes of distress, and mandatory clinician verification before clinical action or documentation. We also examine risks related to suggestibility, bias, medication effects, privacy, alert fatigue, workload, and overreliance on AI-generated outputs. A five-step research pathway is proposed that includes co-design, technical testing, clinical validation, evaluation across populations and care settings, and risk-based governance. Generative AI should be adopted only if prospective studies demonstrate meaningful value beyond validated assessment methods and usual clinical workflows without reducing patient choice, replacing clinical judgment, or increasing workload.

## 1 Clinical vignette: the hidden pain behind agitation in dementia

Mrs. L, an 84-year-old woman with moderate dementia living in long-term care, became increasingly agitated during morning care. Staff documented resistance to bathing, yelling during transfers, and pacing, but initially attributed these behaviors to dementia progression. A bedside evaluation brought together her daughter's observations, movement-related behaviors, physical examination, and clinical history. Osteoarthritis was identified as a possible contributor to her distress. After a monitored pain-management plan and reassessment, her agitation decreased and her participation improved.

This situation is common in clinical practice. Pain and neuropsychiatric symptoms often overlap in older adults with cognitive impairment. Agitation, wandering, aggression, or resistance to care may reflect pain, another source of distress, dementia progression, or more than one of these factors (1, 2). When communication is impaired, clinicians must bring together several sources of information without assuming that any single behavior proves pain. This challenge contributes to underrecognition and undertreatment (3).

Cognitive impairment includes a wide range of abilities, from mild impairment to advanced dementia. A person with mild impairment may still provide a meaningful self-report, while a person with advanced dementia may require greater reliance on observed behavior, caregiver knowledge, and clinical examination. The assessment approach must therefore be matched to the person's cognitive, communication, and sensory abilities.

## 2 Current approaches to pain assessment

Self-report remains the preferred source of pain information whenever it is feasible. Clinicians may need to use simple verbal or categorical response options when abstract numerical scales are difficult to understand. When reliable self-report is not possible, validated observational instruments provide an important clinical foundation. PAINAD, the Abbey Pain Scale, PACSLAC/PACSLAC-II, MOBID-2, and PAIC15 assess overlapping but different behaviors, including facial expression, vocalization, body movement, breathing, consolability, and changes during movement or care (4–6).

These tools also have limitations. Their performance varies by instrument, setting, rater, and observation context. Some tools perform better during movement than at rest, and MOBID-2 has shown responsiveness after a pain-treatment protocol (7, 8). Their feasibility also differs across acute and long-term-care settings (9). Caregivers contribute valuable knowledge of the person's usual behavior, but their reports may differ from patient or staff reports. Repeated within-person observation can strengthen assessment, although no single behavior is specific to pain. Any use of generative AI must therefore add value beyond these established approaches.

## 3 What generative AI could add

Artificial intelligence is already used in pain assessment. Existing approaches include automated classification of facial and behavioral indicators, PainChek, conventional natural language processing of clinical communication, and analysis of EHR notes (10–13). Most detection systems use discriminative AI to classify or score existing data. Generative AI, including large language models, is different because it can respond to language, organize information from several sources, and produce a written or conversational output.

The key question is not whether generative AI can detect pain. Its possible role is to help clinicians review fragmented or conflicting information while keeping the original sources, disagreements, missing information, alternative explanations, and uncertainty visible. A bounded system could organize patient, caregiver, staff, observational-tool, EHR, and medication information and prompt a clinician to reassess the person. Whether this support improves decisions without increasing false alerts, workload, or error remains unknown and must be tested (14, 15).

## 4 A person-centered evidence-to-action framework

The framework (Figure 1) begins with the person, not the technology. Assessment should consider the person's preferences, usual behavior, communication abilities, cognitive and sensory status, assent, dissent, and right to refuse. Evidence may include self-report when feasible, caregiver and staff observations, validated observational tools, physical examination, clinical history, EHR information, medication timing, and changes from the person's usual baseline. These sources may be incomplete or may disagree.

**Figure 1:**
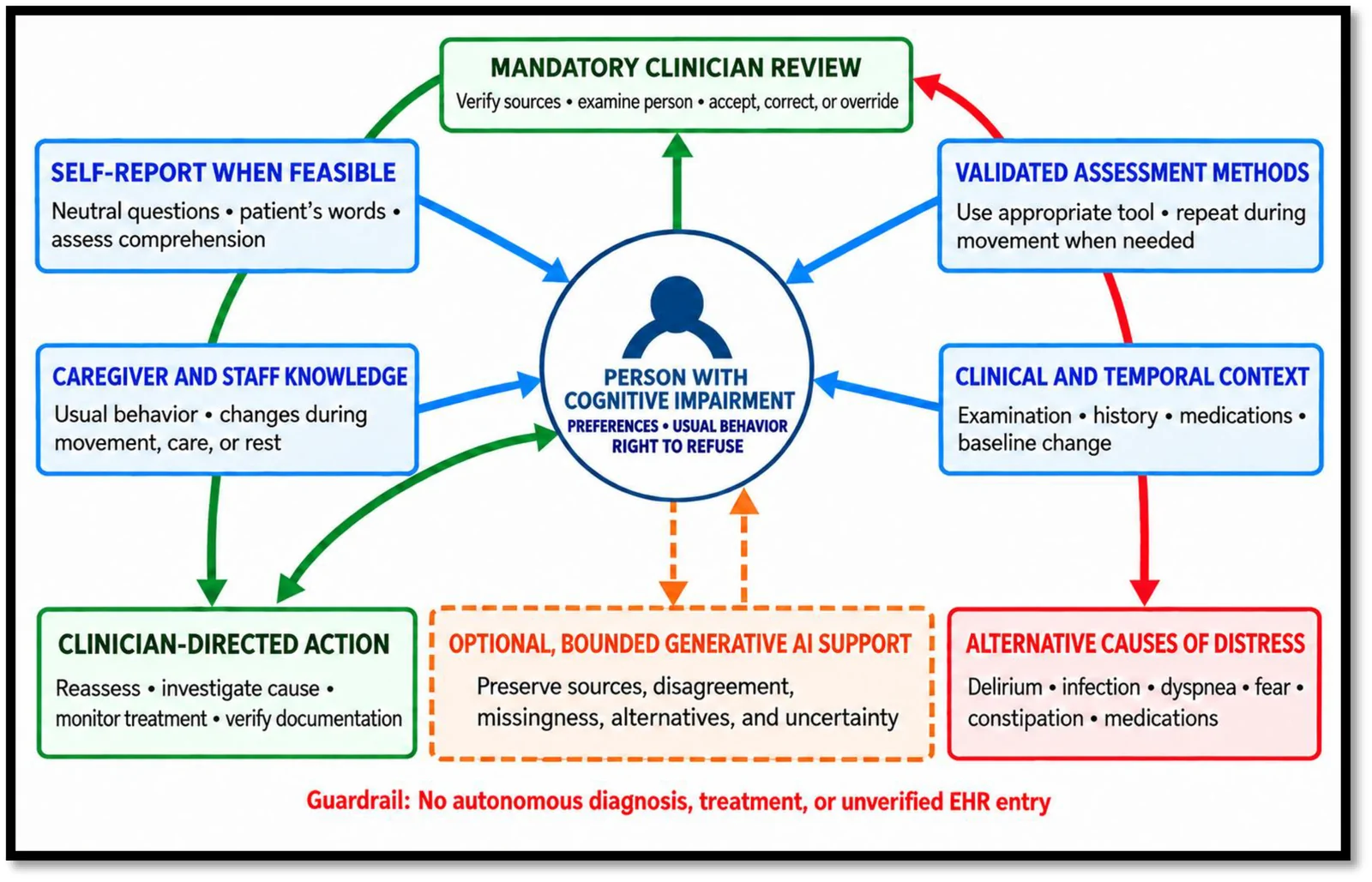
Person-centered, clinician-governed framework for bounded generative AI support in pain assessment. Generative AI may support communication and organize information, but clinician review is required before clinical action. All proposed benefits require prospective testing.

Generative AI is an optional support within this process and is limited by three safeguards. First, only appropriate, source-identified information should enter the system. Consent or assent is required when the system interacts directly with the person. Second, the AI output should separate observation from interpretation, show agreement and disagreement, identify missing information, and present uncertainty and other possible causes of distress. Third, a clinician must verify the information, assess the person, and accept, correct, or override the output before any clinical action or EHR documentation.

Generative AI may be useful when fragmented or conflicting information delays reassessment. It may add little when the available evidence is already clear, when no clinician is available to respond, or when additional alerts increase workload. It should never independently diagnose pain, select treatment, or place an unverified conclusion in the EHR. After any clinical action, reassessment provides new information and returns the process to the person-centered evidence base.

## 5 Three core functions and seven testable use cases

The proposed uses of generative AI can be grouped into three connected functions. Capacity-sensitive elicitation supports communication when the person can participate. Source-attributed synthesis organizes information from the person, caregivers, staff, validated tools, and the clinical record. Longitudinal support and communication identify changes over time and prepare relevant information for clinical review. These functions do not have to be used together. Their use should depend on the person's abilities, the available information, and the clinical setting.

The three functions are complementary but not sequential requirements. Capacity-sensitive elicitation may be used when the person can meaningfully participate. Source-attributed synthesis may then organize the person's responses with caregiver observations, staff reports, validated assessment scores, medication information, and clinical findings. Longitudinal support and communication may identify changes from the person's usual pattern over time. One, two, or all three functions may be used depending on the clinical situation.

For example, a person with mild cognitive impairment may benefit from an adaptive interview and longitudinal diary, whereas a person with advanced dementia may require synthesis of observational, caregiver, and clinical information without direct AI interaction. In all cases, the AI output supports clinician reassessment and does not independently diagnose pain or determine treatment. Table 1 presents seven use cases and shows the user, setting, information entered, expected output, required clinical action, major risks, safeguards, evidence status, and outcomes. These use cases are testable proposals, not claims of benefit.

**Table 1:** Three core generative functions and seven testable use cases in pain assessment.

Use case	User and setting	Inputs and bounded AI output	Human verification and action	Principal harm, safeguard, evidence status, and outcomes
CORE FUNCTION 1: CAPACITY-SENSITIVE ELICITATION				
Adaptive conversational interview	Patient with mild-to-moderate impairment; clinic, home, or calm long-term-care encounter	Neutral scripted questions and comprehension and refusal checks → verbatim transcript, constrained rephrasing, and explicit uncertainty	Clinician confirms understanding and self-report and stops the interaction if comprehension, attention, or assent is inadequate	Suggestibility → locked neutral scripts, limits on rephrasing, and stop rules. Evidence: Conceptual. Outcomes: comprehension, refusal, missingness, discordance, and time.
Possible pain-related language cue identification	Patient or caregiver; clinic, portal, home, or recorded encounter	Speech or text with functional context → possible cue and competing explanations, not a pain label	Clinician asks neutral follow-up questions and evaluates pain alongside other possible causes	False attribution → prespecified thresholds and visible alternative explanations. Evidence: Conceptual. Outcomes: clinically useful follow-up, positive predictive value, false alerts, and subgroup performance.
CORE FUNCTION 2: SOURCE-ATTRIBUTED SYNTHESIS				
Behavioral narrative structuring	Nurse or direct-care worker; long-term or acute care	Source-tagged notes and validated observational-tool items → structured summary linked to the original observations	Nurse checks the summary against the source and conducts bedside reassessment when warranted	Omission or distortion → side-by-side source display and blocked unsupported conclusions. Evidence: Adjacent NLP evidence only. Outcomes: source agreement, correction rate, review time, and missed cues.
Multi-informant synthesis	Clinician; care transitions, team review, acute care, or long-term care	Patient, caregiver, staff, validated tool scores, EHR information, and medication timing → agreement, disagreement, missing-data, and alternative-explanation summary	Clinician reconciles the sources, examines the person, and selects the next assessment or investigation	Amplification of proxy or documentation bias → identify every source and preserves dissent. Evidence: Conceptual. Outcomes: discrepancy detection, correction and override rates, decision latency, and workload.
Synthesis of validated nonverbal indicators	Clinician caring for a person with advanced dementia; long-term or acute care	Validated observational score, within-person baseline, and clinical context → source-linked synthesis with detector confidence, uncertainty, and alternative explanations	Clinician independently examines the person, applies an appropriate assessment, and may correct or override the output; no autonomous treatment	Detector error may be converted into persuasive prose → separate detector confidence and source data from generated interpretation. Evidence: Indirect evidence only. Outcomes: incremental value over the established tool alone, fidelity, corrections, overrides, false alerts, and clinical outcomes.
CORE FUNCTION 3: LONGITUDINAL SUPPORT AND COMMUNICATION				
Longitudinal pain-related diary	Person with early cognitive impairment, with optional caregiver support; home or community setting	Brief symptoms, activity, function, and medication reports → within-person trajectory and prespecified change flag	Clinician checks adherence, missingness, baseline, medication context, and alternative explanations before acting	Surveillance burden and alert fatigue → proportionate sampling, prespecified thresholds, and suppression of low-value alerts. Evidence: Conceptual. Outcomes: adherence, missingness, signal-to-alert ratio, workload, decision impact, pain, and function.
Caregiver coaching and communication support	Family caregiver; home, community, or long-term care	Caregiver observations and approved educational content → neutral observation prompts, structured communication, and escalation guidance	Nurse or clinician reviews submitted observations; the caregiver and system do not diagnose pain	Unsafe advice, documentation bias, or caregiver burden → approved content, separation of observation from interpretation, and escalation limits. Evidence: Conceptual. Outcomes: documentation quality, appropriate escalation, inequitable access, and caregiver burden.

Evidence status refers to direct evidence for the proposed generative function in this population. Evidence for self-report, observational instruments, discriminative AI, or conventional NLP does not establish the effectiveness of the proposed generative component.

### 5.1 Core function 1: capacity-sensitive elicitation

This function is appropriate only when the person can meaningfully participate. It includes adaptive interviews and identification of possible pain-related information in everyday language. The purpose is to reduce communication barriers while preserving the person's own words, understanding, uncertainty, assent, dissent, and right to refuse.

#### 5.1.1 Adaptive conversational pain interviews

Self-report should be attempted whenever feasible. A bounded interview could present one neutral question at a time, repeat rather than steer a question, record the exact wording used, and stop when attention, understanding, or assent is inadequate. It should not continue during marked agitation. Studies should test whether limited adaptation improves comprehension or completeness without increasing suggestibility or disagreement with the person's intended response.

When meaningful interaction is not possible, the system should not attempt to elicit self-report. Its role should shift to organizing clinician and caregiver observations. Silence or refusal must never be interpreted as evidence for or against pain.

#### 5.1.2 Possible pain-related information in everyday language

A language model might identify a statement such as ‘I stopped gardening’ as a reason for neutral follow-up. It should not label the statement as pain because fatigue, fear, depression, dyspnea, cognitive change, or environmental barriers may also explain it. The output is useful only if it supports appropriate follow-up without producing too many false alerts.

### 5.2 Core function 2: source-attributed synthesis

This function organizes information from the person, caregivers, staff, validated tools, and the clinical record without making an independent pain diagnosis. Every summary should identify the source, separate observation from interpretation, show areas of agreement and disagreement, identify missing information, and present uncertainty and other possible causes of distress.

#### 5.2.1 Structuring behavioral observations

Generative AI could convert source-tagged clinical notes into a structured description while keeping the original wording available. For example, ‘winced when standing and pulled away during dressing’ may support movement-focused reassessment, but it does not prove pain. Validation should focus on whether the summary accurately reflects the source and avoids omissions, distortions, or unsupported additions (12).

#### 5.2.2 Multi-informant synthesis

A synthesis tool could organize self-report, caregiver and staff observations, validated scores, clinical conditions, medication timing, and changes over time. It should identify who reported each observation and what remains unknown. The clinician would decide whether to examine the person, repeat a validated assessment, investigate another cause of distress, continue observation, or take no further action.

#### 5.2.3 Synthesis of validated nonverbal indicators

Nonverbal pain detection is an established use of discriminative AI, not a new generative function (16). PainChek combines automated facial analysis with structured observational indicators and has been evaluated in people with dementia. PainChek Adult received FDA *de novo* classification in 2025 (10, 17–19).

A generative component should be added only if it improves the validated detector or usual workflow. It might organize detector confidence, observational scores, baseline behavior, medication timing, and caregiver observations while showing uncertainty and other explanations. However, a fluent summary could make an incorrect detector output appear more certain. Detector accuracy must therefore be evaluated separately from the accuracy and usefulness of the generated summary. Within-person change may be informative, but it is not specific to pain and should prompt further assessment rather than automatic treatment.

### 5.3 Core function 3: longitudinal support and communication

This function organizes information collected over time and prepares relevant observations for professional review. Its purpose is to identify changes from the person's usual pattern and improve communication, not to treat a change as proof of pain.

#### 5.3.1 Longitudinal pain-related diaries

People with early cognitive impairment, with optional caregiver support, could use brief voice or text diaries to report symptoms, activity, function, and medication use. The system could summarize within-person patterns and identify prespecified changes. It should also show missed entries and allow clinicians to suppress repetitive or low-value alerts. Evaluation should include adherence, burden, false alerts, clinical decisions, staff workload, pain, and function.

#### 5.3.2 Caregiver coaching and communication support

Caregiver-facing tools could provide approved education, neutral observation prompts, structured communication support, and guidance about when to seek professional evaluation. They should not diagnose pain or provide medication advice. Studies should examine caregiver workload, access, documentation quality, appropriate escalation, and whether the system increases or reduces existing bias.

## 6 How the three functions could apply to Mrs. L

In Mrs. L's case, direct AI conversation may not be appropriate during agitation. Instead, source-attributed synthesis could bring together her daughter's report, staff observations during transfers and bathing, the observational-tool score, physical examination findings, and medication timing. Longitudinal support could show that these behaviors were new or occurred mainly during movement. The output would not diagnose or treat pain. It would prompt the clinician to assess Mrs. L, consider pain, and other causes of distress, and decide on the next step. This example shows that the three functions are selected according to the person's condition; they are not automatically used together.

## 7 Ethical, clinical, and implementation considerations

Risks depend on how the system is used. A model may incorrectly interpret delirium, dyspnea, fear, constipation, infection, environmental overstimulation, or medication-related sedation as pain. Leading questions may change self-report, biased caregiver documentation may be amplified, and an unsupported summary may be copied into the EHR. Every output should retain its sources, alternative explanations, uncertainty, and an audit trail. Autonomous EHR entry and unverified diagnostic language should be blocked.

Bias and equity should be assessed across race, ethnicity, language, sex, education, sensory status, dementia type and stage, and care setting. Findings from one model, prompt, or clinical example cannot establish fairness (20). Studies should report subgroup errors, missing information, refusal, and alert burden, and systems should be monitored after implementation.

Direct interaction requires safeguards. These include assessment of the person's ability to participate, consent or assent, clear disclosure that the system is not human, neutral scripts, limits on repetition and rephrasing, an easy way to stop, and referral to a clinician when understanding is uncertain. Clinicians should be able to review the exact prompts and responses. Audio or video capture requires a clear purpose, consent procedures, limited data collection, access controls, and retention limits (15, 21, 22).

Clinician review is an active step, not simply a disclaimer. The clinician must confirm the sources, assess the person, consider other causes of distress, use an appropriate validated tool, and accept, correct, or override the output. Responsibility for assessment and treatment remains with qualified healthcare professionals.

Medication timing must also be considered. Antipsychotics, benzodiazepines, antidepressants, opioids, anticholinergic medications, and other sedating drugs may change attention, speech, facial expression, movement, and agitation. Systems should include dose and timing when available and should be tested for medication-related errors (23).

Implementation should be evaluated in the actual clinical workflow. Poorly targeted prompts may increase alert fatigue, duplicate documentation, or shift work to frontline staff (24). Studies should measure review time, alert volume, correction and override rates, staffing and training needs, interoperability, cost, and whether a clinician is available to respond.

## 8 Future research directions

A clear validation pathway is needed before clinical use. The pathway should proceed in five steps.

### 8.1 Step 1 define and co-design the use case

Researchers should identify the intended user, care setting, information entered, expected output, clinical decision supported, and actions the system must not take. People living with cognitive impairment, caregivers, nurses, physicians, direct-care workers, informaticians, and regulators should help define usability, acceptable workload, safeguards, and stopping rules.

### 8.2 Step 2 complete technical testing

Testing should determine whether the system follows its instructions, produces consistent results, identifies each information source, and accurately reflects the original observations. It should also assess unsupported additions, communication of uncertainty, privacy, and performance in difficult clinical scenarios.

### 8.3 Step 3 establish clinical validity

Studies should compare the full AI-supported workflow with usual care, self-report when feasible, validated observational tools, and expert clinical assessment. Because advanced dementia has no single gold standard for pain assessment, validation should combine clinical examination, known painful conditions, or activities, repeated within-person observation, caregiver knowledge, and monitored response to treatment. Improvement after analgesic treatment should not be treated as proof that pain was present (25).

### 8.4 Step 4 test across populations and care settings

External and prospective studies should include different levels of cognitive impairment, languages, demographic groups, sensory impairments, and care settings. Studies should measure clinician corrections and overrides, false alerts, time to clinical action, treatment changes, pain, function, adverse events, caregiver burden, and staff workload. The main test is whether generative AI adds meaningful value beyond validated tools and usual clinical workflows, not whether the model performs well by itself.

### 8.5 Step 5 match governance to clinical risk

Regulatory requirements should reflect the system's intended use. A tool that only organizes documentation presents less risk than one that prioritizes reassessment, interprets medical-device information, or recommends treatment. The degree of autonomy, clinical claims, device integration, system updates, and influence on treatment will determine the relevant FDA pathway and EU AI Act requirements (19, 26). Human-factors testing, audit trails, incident reporting, change controls, and workforce education should be matched to the level of risk.

## 9 Conclusion

Generative AI is not needed to observe, classify, or score pain behaviors. Its possible role is to organize information while keeping the sources, disagreements, alternatives, and uncertainty clear. It should be used only if it adds value beyond existing assessment without replacing clinical judgment or adding excessive workload. Until prospective studies show patient benefit, these claims remain hypotheses.

## Data Availability

The original contributions presented in the study are included in the article/Supplementary Material, further inquiries can be directed to the corresponding author.
